# Microwave Near Field Imaging of Externally Injected Signals in an Encapsulated Electronic Device

**DOI:** 10.3390/mi17060711

**Published:** 2026-06-10

**Authors:** Qiang Zhu, Yangfan Zhang, Xin Li, Huanfei Wen, Jun Tang, Jun Liu

**Affiliations:** 1 State Key Laboratory of Widegap Semiconductor Optoelectronic Materials and Technologies, North University of China, Taiyuan 030051, China; 20240039@nuc.edu.cn (Q.Z.); zhangyf0134@163.com (Y.Z.); 2 State Key Laboratory of Extreme Environment Optoelectronic Dynamic Testing Technology and Instrument, North University of China, Taiyuan 030051, China; 3 Science and Technology on Electronic Test and Measurement Laboratory, North University of China, Taiyuan 030051, China; wenhuanfei@nuc.edu.cn

**Keywords:** quantum sensor, non-destructive testing, USB flash drive, NV center

## Abstract

Addressing the challenges of electromagnetic compatibility testing and non-destructive inspection of internal structures in miniaturized electronic devices. This paper reports a non-destructive testing method based on wide-field imaging using diamond nitrogen-vacancy (NV) centers, and systematically demonstrates its application on a black-epoxy-encapsulated Universal Serial Bus (USB) flash drive. In the experiment, a swept microwave signal from 2.82 GHz to 2.97 GHz was sequentially injected into the four external interface pins of the USB drive. A bulk diamond served as the quantum sensing layer, and optically detected magnetic resonance (ODMR) was employed to perform wide-field imaging of the microwave field distribution on the surface of the signal lines within a 1 × 1 mm^2^ region of interest. The experimental results show that the microwave field distributions corresponding to different interface channels are significantly different. Based on these differences, the connection relationship between each signal line and its corresponding interface pin can be clearly identified, and the differences in field distribution as well as crosstalk characteristics among channels can be revealed. The method established in this work provides an effective technical pathway for non-destructive electromagnetic testing and functional verification of electronic products.

## 1. Introduction

Currently, portable storage devices represented by USB flash drives have become the core medium for information transmission and storage [1]. Their signal integrity, interface interconnection reliability, and internal wiring quality directly affect data security and user experience. However, conventional electrical testing methods, such as time-domain reflectometry (TDR), continuity testing, and impedance analysis, can only obtain lumped parameter information through external interfaces and cannot provide the spatial distribution of internal signal lines or their correspondence with the interfaces. The fully encapsulated physical structure of black-epoxy USB flash drives makes it difficult to perform in situ observation of internal microwave signals using traditional electrical probing methods [2]. Therefore, a non-destructive microwave field characterization technique is required for functional verification of internal circuits at the electronic product level.

Over the past few decades, significant progress has been made in various quantum microwave measurement techniques, including scanning microwave microscopy [3], superconducting quantum interference devices [4], Rydberg atoms [5,6], and diamond nitrogen-vacancy (NV) center sensors. Among them, the NV center in diamond—a point defect with a room-temperature stable spin state—has shown broad application prospects in the fields of magnetic fields [7], electric fields [8,9,10], temperature [11,12,13], strain [14], and biological imaging [15]. In addition, the optically detected magnetic resonance technique based on NV centers enables high-resolution imaging of microwave magnetic fields from the nanometer to millimeter scale under ambient conditions. With the advantages of all-optical and non-invasive operation, this technique has been widely applied in near-field microwave measurement and chip-level electromagnetic compatibility testing [16]. For example, companies such as Quantum Diamonds and DiaSense are advancing toward commercialization based on NV center technology. Their core approach involves measuring the stray magnetic fields generated by currents inside devices, using NV magnetometry to achieve failure analysis and defect localization in semiconductor chips, primarily targeting wafer-level or chip-level magnetic imaging. In contrast, this paper focuses on near-field microwave electric field imaging, directly detecting the microwave electric field distribution radiated by signal lines inside packaged devices.

Building on this previous work [17], this paper further extends the high-fidelity wide-field microwave imaging method with NV concentration compensation to characterize the surface microwave field of a practical electronic device—namely, a black-epoxy USB flash drive. By sequentially injecting swept microwave signals into the four external interface pins of the USB drive, the microwave field distribution over a 1 × 1 mm^2^ region of the device’s circuitry is imaged using diamond NV centers. This technique shows great potential for investigating internal circuitry in a non-destructive manner at the electronic product level.

## 2. Experimental System and Diamond

**Figure 1:**
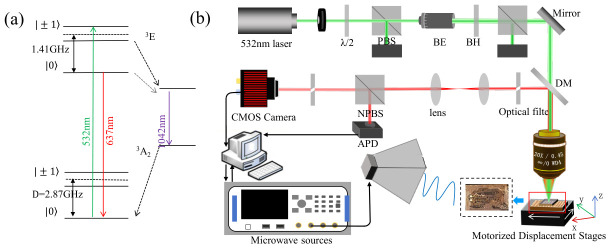
(**a**) Energy level diagram of the NV center. (**b**) Schematic diagram of the experimental setup. BE: beam expander; BH: beam homogenizer; PBS: polarizing beam splitter; DM: dichroic mirror; NPBS: non-polarizing beam splitter.

The three-level system [18] of the diamond NV center is shown in Figure 1a, consisting of the ^3^A_2_ ground state, the ^3^E excited state, and a metastable state. Using 532 nm green laser excitation, the NV center can be excited to the excited state and then relaxes back to the ground state, emitting fluorescence in the phonon sideband around 680 nm. Owing to intersystem crossing (ISC) [19,20], the excited $\left|\left.\pm 1\right\rangle \right.$ sublevel preferentially decays to the metastable state via non-radiative processes and then returns to the $\left|\left.0\right\rangle \right.$ ground state. The $\left|\left.\pm 1\right\rangle \right.$ state has a long lifetime (~300 ns), resulting in a fluorescence intensity reduction of about 30% compared to the $\left|\left.0\right\rangle \right.$ state. Thus, the spin state of the NV center can be distinguished by measuring the change in fluorescence power, which forms the basis of optical spin readout. In the absence of an external magnetic field, the $\left|\left.\pm 1\right\rangle \right.$ sublevels are degenerate [21] and exhibit a zero-field splitting of 2.87 GHz from the $\left|\left.0\right\rangle \right.$ state. When an external magnetic field is applied, the $\left|\left.\pm 1\right\rangle \right.$ energy levels undergo Zeeman splitting, and the resonant microwave frequency shifts with the magnetic field while remaining centered at 2.87 GHz. By sweeping the microwave frequency and detecting the ODMR fluorescence signal, the field strength can be determined.

The experimental system of this study is shown in Figure 1b. A 532 nm laser (MLL-F-532-1W, amplitude noise < 1% (rms, 20 Hz–20 MHz), beam diameter 1.2 mm, Changchun New Industries Optoelectronics Technology Co., Ltd., Changchun, China) emitted the laser beam, which passed through a beam expander (OSE05-532, JCOPTIX, Nanjing, China) and a beam homogenizer (DOE25H-532-6-FTS50, operating wavelength 532 nm, LBTEK, Shenzhen, China) to be converted into a flat-top beam that fully filled the objective lens. The beam was then focused onto the USB flash drive through a 20× objective lens (numerical aperture NA = 0.45). The collection optical path consisted of a photodetector (THORLABS, Newton, NJ, USA, APD4303A2/M), a Complementary Metal Oxide Semiconductor (CMOS) camera (THORLABS, Newton, NJ, USA, CS505MU), and other optical components. In the experiment, the USB drive was fixed on a motorized translation stage, and a diamond was placed in close contact with its surface to serve as the sensing layer for reading the near-field microwave field strength signals of the USB drive. In this system, the USB drive under test itself acted as the active microwave emission source, eliminating the need for an external microwave radiation antenna (e.g., a horn antenna [22,23]) and thus avoiding interference from non-uniformities in the external microwave field.

The tested sample is a commercially available black-epoxy USB flash drive (128 MB), whose encapsulated interior contains multiple signal lines, with four input interface pins exposed on the outside of the encapsulation. During the experiment, SMA connectors were soldered to the four exposed pins of the USB drive. These four pins were then connected to a signal generator (frequency sweep range: 9 kHz–40 GHz, power adjustable from −144 dBm to 30 dBm, Agilent N5183B, Santa Clara, CA, USA). The swept microwave signal was sequentially coupled into each interface channel during the imaging process.

**Figure 2:**
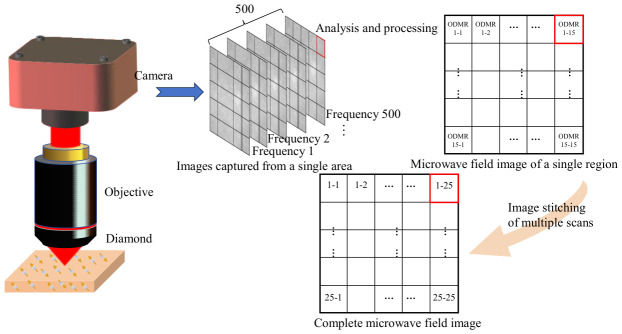
Flowchart of microwave field imaging on the surface of the USB flash drive.

The frequency hopping of the microwave source and the exposure triggering of the CMOS camera were synchronously controlled by the same clock source (signal generator), ensuring that each image frame strictly corresponded to a specific operating frequency. During the experiment, as shown in Figure 2, the motorized translation stage moved the USB drive in steps of 40 μm. For a single measurement, each pixel of the Thorlabs camera could be treated as an independent photodetector. By performing Lorentz fitting on the data from each pixel of the images acquired over a single imaging region, the ODMR contrast was extracted. The value obtained for each pixel was then mapped back to that pixel, yielding the microwave field imaging result for that region. Finally, the microwave field intensity images of each region were stitched together according to the scanning path to obtain a complete microwave field intensity distribution image, thereby completing the microwave field imaging of the scanned area (1 × 1 mm^2^).

The diamond used in this study was fabricated using the high-pressure high-temperature (HPHT) method [24] and subsequently subjected to electron irradiation with a dose of 9.8 × 10^18^ cm^−2^ using 10 MeV electrons [25], followed by vacuum annealing at 850 °C for 2.5 h. In our previous work [17], the influence of NV concentration was calibrated for this diamond. On this basis, the systematic imaging error introduced by NV concentration inhomogeneity could be eliminated, ensuring the reconstruction of the true microwave field intensity distribution.

## 3. Results and Discussion

The contrast and linewidth of the ODMR signal are closely related to the microwave amplitude, indicating that they can be used to reconstruct the microwave field intensity distribution. To obtain the ODMR information for each pixel in the imaging region, a Lorentzian function was used for fitting, as expressed in the following equation:

(1) $$W\left(C,\delta_{v}\right)=A_{0}+\frac{2}{\pi }\left(\frac{A\delta_{v}}{4{\left(X-X_{c}\right)}^{2}+{\delta_{v}}^{2}}\right)$$

where $A_{0}$ is the fluorescence intensity at the off-resonant frequency in the ODMR spectrum, A is the area of the resonance peak, $\delta_{v}$ is the full width at half maximum (FWHM),$\;X_{c}$ is the resonance frequency, X is the frequency at the fitting point, and $C=-2A/(\pi A_{0}\delta_{v})$ is the ODMR contrast. According to Ref. [26], the microwave field intensity at each pixel can be calculated as:

(2) $$B_{⊥}=\left|B\times n\right|\approx \frac{\sqrt{2}\delta_{v}}{\gamma_{e}}\sqrt{\frac{C}{C_{max}}}$$

where $C_{\max}$ represents the saturated optical contrast. Equation (2) has two important limiting cases. When the microwave power is above the saturation threshold, due to power broadening, the linewidth $\Omega \approx 1/\sqrt{2}\gamma_{e}B_{⊥}$ is proportional to $B_{⊥}$. For low microwave power, $\delta_{v}$ remains constant, while the optical contrast $C\propto B_{⊥}^{2}$ is proportional to the absorbed microwave power. That is, the ODMR contrast (the fractional decrease in fluorescence at resonance) increases with increasing microwave power until saturation is reached, and its variation curve depends on the ratio of the microwave field strength to the spin relaxation rate. In our imaging experiments, the laser power was kept constant and in saturation, and a microwave power of 5 dBm was used to ensure weak driving conditions. In our previous work [17], the linear relationship between microwave power and contrast was analyzed and verified.

**Figure 3:**
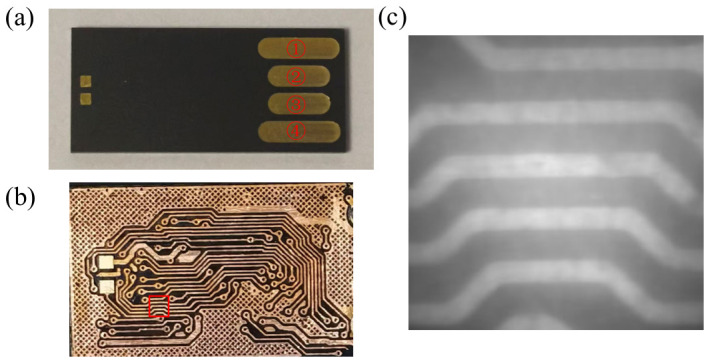
(**a**) Photograph of the black-epoxy USB flash drive. (**b**) USB drive with exposed circuitry after processing, where the red box indicates the measurement region. (**c**) Grayscale image of (**b**) captured by the CMOS camera.

As shown in Figure 3a, the four interfaces of the measured black-epoxy USB flash drive are, in order, the power supply positive terminal, the data line negative terminal, the data line positive terminal, and the ground terminal. During the experiment, the frequency of the swept microwave signal was set from 2.82 GHz to 2.97 GHz with a step of 400 kHz, and the microwave power was set to 5 dBm. The swept microwave was sequentially injected into the four interfaces. The area inside the red box in Figure 3b was selected as the complete measurement region. In the CMOS camera image (Figure 3c), we observed six signal lines in this region, whose internal connections to the four external interfaces of the USB drive were unknown. It should be noted that the measurement object during our experiment was the USB drive shown in Figure 3a, i.e., a brand-new, fully functional USB drive. Figure 3b,c were obtained after the experiment for verification purposes.

**Figure 4:**
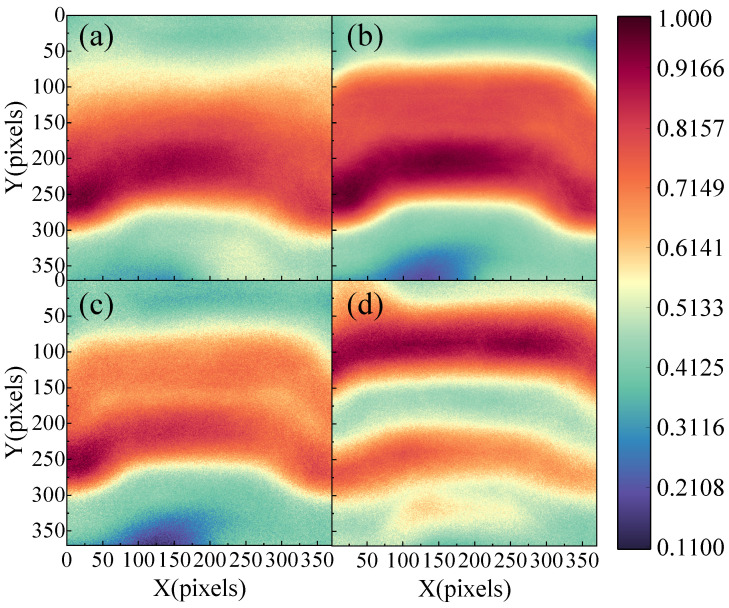
Microwave field imaging results on the surface of the USB flash drive: (**a**–**d**) correspond to the results when microwave signals are injected into interfaces 1–4, respectively. The field intensity is normalized.

As shown in Figure 4, the microwave imaging results after concentration-compensation correction clearly reveal the connections between the microstrip lines of the USB flash drive and the external interfaces within the selected region. Figure 4a shows that when a microwave is injected into Interface ①, the region with stronger field intensity covers signal lines 2, 3, and 4, with the strongest radiation occurring between lines 3 and 4, while the field intensity at signal line 2 is weaker and exhibits a radiative pattern, indicating that the excitation source for Interface ① is mainly located at signal lines 3 and 4, although a measurable field intensity is also generated at line 2 due to electromagnetic diffusion. In Figure 4b, after microwave injection into Interface ②, the field intensity is also concentrated in the region of lines 2–4, with the radiation intensity between lines 3 and 4 being even stronger than that for Interface ①. Meanwhile, the upper edge of signal line 2 forms a clear field intensity boundary, indicating that the excitation source for Interface ② corresponds to signal lines 2, 3, and 4, with line 4 being the strongest. Figure 4c corresponds to microwave injection into Interface ③, where the field intensity distribution shows equal intensity in the regions between lines 2–3 and between lines 3–4, while the field intensity directly at signal line 3 is relatively weak. This indicates that the signal line driven by Interface ③ is line 3, with its microwave field symmetrically distributed in the near-field regions on both sides of line 3. In Figure 4d, after microwave injection into Interface ④, the field intensity reaches its maximum at signal line 2, while a clear signal is also observed at line 4, and a weak radiation appears at line 5, indicating that Interface ④ is primarily connected to line 2 and couples via crosstalk to signal lines 4 and 5.

Therefore, we can infer that Interface ① is connected to the lines in the 3–4 region, Interface ② is connected to the same region but with a more concentrated near field, Interface ③ is connected to signal line 3, and Interface ④ is connected to signal line 2 with an auxiliary ground. The above results verify the capability of NV-based wide-field microwave imaging to distinguish internal connections and crosstalk in USB flash drives. Since the concentration compensation has removed the systematic inhomogeneity of the diamond itself, and because no metal wires were exposed due to the USB drive encapsulation, the influence of non-uniform excitation light intensity caused by metal wire reflections in previous work is absent. The experimental results obtained in this study well reflect the microwave field distribution characteristics radiated by the internal signal lines of the black-epoxy USB flash drive and allow inference of their connections to the external interfaces.

## 4. Conclusions

In this paper, we propose a non-destructive testing method for electronic devices based on wide-field microwave imaging using diamond NV centers. Using a black-epoxy USB flash drive as a practical sample, we experimentally demonstrated the entire process from microwave injection and fluorescence acquisition to microwave field reconstruction. By sequentially applying swept microwave signals to the four external interfaces of the USB drive and performing wide-field imaging of the signal lines in a fixed region, we successfully obtained the spatial microwave field distribution maps near the signal lines on the USB drive surface. The imaging results not only clearly reveal the morphology and radiation characteristics of the signal lines corresponding to each interface channel but also intuitively present the crosstalk among multiple signal channels. The findings of this work validate the feasibility of NV-center-based wide-field microwave imaging for non-destructive testing and internal functional verification of electronic products. It should be noted that the signal injected in this experiment is a continuous swept microwave signal, rather than an actual USB data communication signal. Nevertheless, this method still provides a new technical approach for near-field characterization and electromagnetic compatibility diagnostic evaluation of complex electronic systems.

## Data Availability

The data that support the findings of this study are available from the corresponding author upon reasonable request.
